# Influence of the Electrolyte Salt Concentration on DNA Detection with Graphene Transistors

**DOI:** 10.3390/bios11010024

**Published:** 2021-01-17

**Authors:** Agnes Purwidyantri, Telma Domingues, Jérôme Borme, Joana Rafaela Guerreiro, Andrey Ipatov, Catarina M. Abreu, Marco Martins, Pedro Alpuim, Marta Prado

**Affiliations:** 1 Food Quality and Safety Group, International Iberian Nanotechnology Laboratory (INL), Av. Mestre José Veiga, 4715-330 Braga, Portugal; joana.guerreiro@inl.int (J.R.G.); andrey.ipatov@inl.int (A.I.); marta.prado@inl.int (M.P.); 2 2D Materials and Devices Group, International Iberian Nanotechnology Laboratory (INL), Av. Mestre José Veiga, 4715-330 Braga, Portugal; telma.domingues@inl.int (T.D.); jerome.borme@inl.int (J.B.); 3 Nanomedicine Group, International Iberian Nanotechnology Laboratory (INL), Av. Mestre José Veiga, 4715-330 Braga, Portugal; catarina.abreu@i3bs.uminho.pt; 4 Nano-ICs Group, International Iberian Nanotechnology Laboratory (INL), Av. Mestre José Veiga, 4715-330 Braga, Portugal; marco.martins@inl.int; 5 Center of Physics, University of Minho, 4710-057 Braga, Portugal

**Keywords:** liquid gate, graphene, Debye length, phosphate buffer (PB), phosphate buffer saline (PBS), salts, DNA

## Abstract

Liquid-gated Graphene Field-Effect Transistors (GFET) are ultrasensitive bio-detection platforms carrying out the graphene’s exceptional intrinsic functionalities. Buffer and dilution factor are prevalent strategies towards the optimum performance of the GFETs. However, beyond the Debye length (λD), the role of the graphene-electrolytes’ ionic species interactions on the DNA behavior at the nanoscale interface is complicated. We studied the characteristics of the GFETs under different ionic strength, pH, and electrolyte type, e.g., phosphate buffer (PB), and phosphate buffer saline (PBS), in an automatic portable built-in system. The electrostatic gating and charge transfer phenomena were inferred from the field-effect measurements of the Dirac point position in single-layer graphene (SLG) transistors transfer curves. Results denote that *λ_D_* is not the main factor governing the effective nanoscale screening environment. We observed that the longer *λ_D_* was not the determining characteristic for sensitivity increment and limit of detection (LoD) as demonstrated by different types and ionic strengths of measuring buffers. In the DNA hybridization study, our findings show the role of the additional salts present in PBS, as compared to PB, in increasing graphene electron mobility, electrostatic shielding, intermolecular forces and DNA adsorption kinetics leading to an improved sensitivity.

## 1. Introduction

Biosensors for biomolecular detection, such as ion-sensitive field-effect transistors (IS-FETs) for DNA detection, have significantly progressed in recent years as a non-laborious, label-free, and sensitive approach, offering flexibility towards integration with other electronic components, such as signal output reader and data interpreter. Among candidate 2D materials to act as sensing and transducing surfaces in biosensors, graphene is spotlighted for its paramount properties of outstanding electrical conductivity (1738 Siemens/m) [1], biocompatibility, high surface-area-to-volume ratio (2630 m^2^/g), excellent mechanical strength (about 1100 GPa elastic modulus), unparalleled thermal conductivity (5000 W/m/K) [2,3], ambipolar nature from alterable number and type of carriers by gate electric field [4], and the possibility to be incorporated with standard complementary metal-oxide-semiconductor (CMOS) fabrication process [5]. Moreover, with its transparency and low cost, and low environmental impact, graphene is suitable as a sensing material urgently demanded nowadays in various detection schemes. Notably, the wafer-scale single-layer graphene (SLG) grown by chemical vapor deposition (CVD), using copper as a catalyst, has shown remarkable properties as sensing material [6,7,8,9]. In biosensor applications, myriads of strategies have been reported for a wide range of applications. Zheng et al., developed a reusable sensor from directionally transferred CVD graphene for PNA-DNA interaction with the detection limit of ~10 fM for target DNA. Recently, Tsang et al., reported tailoring monolayer CVD graphene for the specific label-free detection of exosomes down to at least 0.1 μg/mL [10]. Meanwhile, also in recent literature, Hwang et al., demonstrated a robust nano-modification of CVD graphene by surface crumpling for DNA detection, which eminently improved the ionic screening area [11]. In our previous work, the CVD-grown SLG was proven excellent in the DNA hybridization detection study down to attomolar concentrations and in signal distinction for a single mismatch in the DNA sequence, i.e., a single nucleotide polymorphism (SNP), on a FET system [12].

The graphene liquid-gated FET’s attractiveness lies in its simple operation, which applies a drop of buffer acting as a gate onto the modified substrate. The sensing area is generally defined by the patterned source (S) and drain (D), and the system typically requires lower gate potential (V_GS_) compared to the back-gated FET that involves the application of a much higher gate voltage [13]. In this particular top-gating mode, the transistor channel’s electrostatic control and the biomolecular interacting sites are naturally obtained by using the electrolyte solution covering the channel area as gate dielectric [14]. Another advantage of this architecture is the high capacitance per unit surface area determined by the electrical double layer (EDL) region formed at the solid-liquid electrolyte interface [15,16]. The high capacitance allows the transistor’s operation at low applied voltage (<1 V), with a low leakage current. It also preserves biomolecules from oxidation and other chemical reactions and their bioactive conformation. The immobilization of a biomatrix, such as DNA onto the graphene FET channel, is regarded as a complex and disturbing interaction that may change the graphene properties and depend on graphene amount of defects [17] and the water-absorbing capacity of the graphene/biomatrix interface [18]. The challenges in liquid gate devices mostly arise from the variety of working buffer compounds and salt ratio. Only a narrow range provides an optimized and ideal interfacial sensing environment.

Electronically, the use of liquid-gated graphene FET is advantageous due to a high transconductance and considerably low noise [19,20]. However, the graphene/electrolyte interfacial characterization is so far still elusive and multi-interpreted. In the electrolytic FET-based operation, the buffer’s proper choice as the background solution is pivotal to maintaining the stable pH upon introducing charged species onto the interface, mostly relying on buffer and salt’s appropriate chemical composition. Phosphate buffer saline (PBS) is the most commonly used buffer in DNA detection and other biological experiments. Its osmolarity and ion concentration are highly similar to that of physiological human body fluids [21]. Significant in FET-based biosensors is the presence of counterions from the background buffer affecting the Debye length (λ_D_), which defines the volume where charges are not entirely electrically screened, forming a space-charge layer [22]. The λ_D_ is too solution composition-dependent [23,24], and therefore, the dilution of buffer solution is commonly employed to increase it. The dilution aims to amplify signals by reducing counterions at the interface’s closest proximity and increasing λ_D_ [25]. Although the dilution strategy has been implemented in formerly reported works [22,26], the underlying mechanism for a highly specific and sensitive biomolecular detection on a liquid-gated FET is not fully understood and rarely investigated.

In this study, we discuss the effects of background electrolyte dilution along with the challenges and trade-off effects on the type of buffer, ionic strength, pH, and the presence of salts, on the limit of detection (LoD), dynamic range, and biomolecular immobilization and binding kinetics in DNA detection. The DNA hybridization detection was performed in a portable and real-time kinetic screening, integrating the multi-array GFET chip with a miniaturized Arduino output reader and an automatic micro-pump. Besides the importance of the ionic strength, we emphasized the significance of using PBS background in DNA detection for the interfacial charge-to-charge interaction on a FET system.

## 2. Materials and Methods

### 2.1. Materials

The PB solution components including Na_2_HPO_4_ and NaH_2_PO4; PBS tablet, NaCl and MgCl_2_ for hybridization buffer mixture, 1-Dodecanethiol (DDT) for Au layer passivation, ethanolamine (ETA) blocking agent, and 1-pyrene butyric acid succinimidyl ester (PBSE) for graphene surface linker and other solvents, such as acetone, ethyl acetate, were purchased from Sigma−Aldrich. A MilliQ system provided the deionized water used in the entire experiment (resistivity at 25 °C = 18.2 MΩ cm). Synthetic oligonucleotides representing the specific DNA from the most common grape varieties in Portuguese wines were used in the hybridization study. The 25-mer sequence of the probe DNA (pDNA) with 3′ C7-amino modification (5′- TCA TAA CCG GCG AAA GGC TGA AGC T-3′) and the complementary target DNA (tDNA) sequence of 5′-AGC TTC AGC CTT TCG CCG GTT ATG A-3′) were synthesized by Metabion International AG. Copper foil with high purity (>99.99%) to assist the graphene growth was purchased from Alfa Aesar or Goodfellow.

### 2.2. Apparatus

Confocal Raman spectroscopy using a WITec Alpha300 R instrument equipped with a Zeiss microscope set of lenses is performed to characterize the graphene channel before using the entire study’s GFET chip. The determination of the Dirac point through the graphene transfer curve is obtained by plugging-in the GFET chip into the built-in credit-card-size Arduino board consisting of a microcontroller, 16-bit digital to analog converters (DAC), 8-bit digital potentiometer, and resistance-controlled current source of 1–100 µA and complementary metal-oxide-semiconductor (CMOS) matrices. The setup is connected to a desktop computer to monitor sensor arrays’ output signal up to twenty transistors. An automatic micropump of a microfluidic system is incorporated for the biomolecular binding kinetic study of the DNA in different electrolytes. A PDMS chamber fitting the layout of the chip with 7 mm^2^ and 0.5 mm height is used to conduct a dynamic flow onto the sensor’s surface. The inlet dimension is 0.5 × 0.5 × 9 mm^3^, while the outlet is 0.5 × 0.5 × 6 mm^3^.

### 2.3. Methods

#### 2.3.1. GFET Chip Fabrication and Surface Characterization

**Figure 1:**
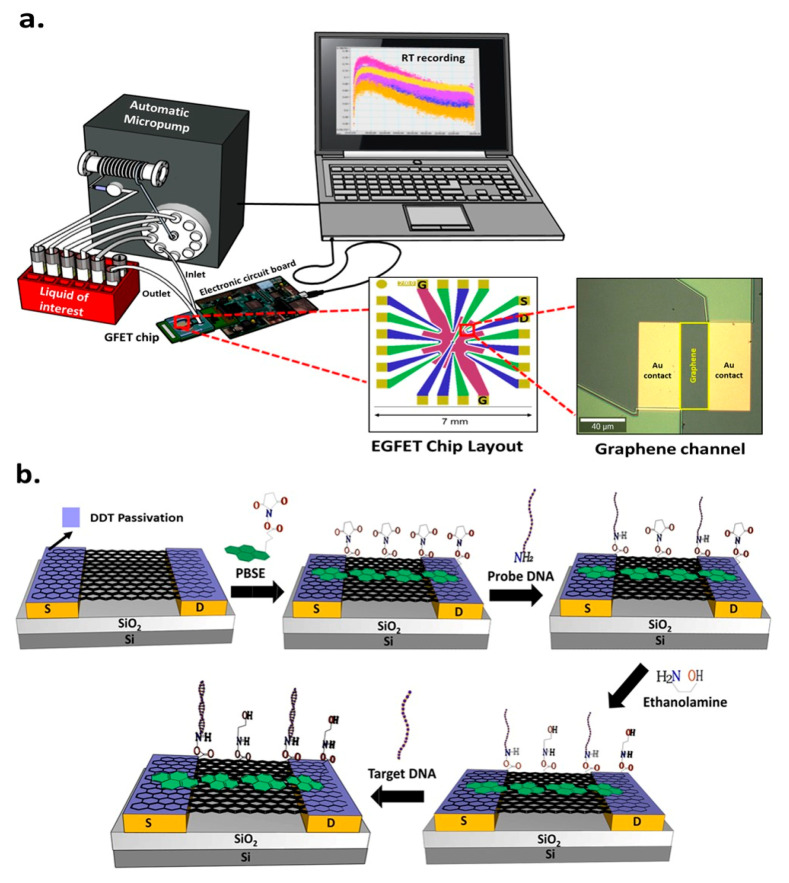
(**a**) The integrated portable system with automatic micropump and electronic reader of the graphene channel on the chip, (**b**) surface functionalization and DNA hybridization strategy.

GFET chip is fabricated on a 200 mm Si wafer (B-doped, 8–30 Ω) covered with 200 nm SiO_2_. A 5 nm Cr was used as an adhesion layer for 40 nm Au deposition. The source (S), drain (D), and gate (G) were patterned using photolithography. A multi-layer of SiO_2_ and 250 nm Si_3_N_4_ was patterned with reactive ion etching (RIE) for current lines passivation. The process was then continued with the single-layer graphene transfer described elsewhere [12] onto the wafer surface with uniform coverage. The wafer with the pattern was diced into multiple equal-sized chips. Each chip consists of one array of 9 graphene sensors on a planar architecture with a receded gate. The source and drain contacts defined the 25 × 75 µm^2^ graphene channel, and the gate diameter was 2.8 mm. The layout of the chip is depicted in Figure 1a. Every single chip is wire bonded and glued with silicone onto a PCB for further electronic reading in the portable platform.

GFET surface characterization is done by Raman spectroscopy to assess the graphene channel quality. The measurement is performed at room temperature in a backscattering geometry with 532 nm and 633 nm lasers excitation using a confocal Raman microscope at an output power of 1.5 mW and an objective ×50 lens, with a numerical aperture of 0.7. A 600 groove/mm grating for three acquisitions under 10 s acquisition time is set to collect the Raman spectra at different random points of the graphene channel to assure the graphene’s uniformity as also checked by Raman imaging.

#### 2.3.2. The Study of the Effects of Ionic Strength and pH

The performance of solution-gated graphene FETs can be affected by the content and the electrolyte solutions’ concentration. We performed the following experiments to study ionic strength’s effect on the solution-gated graphene FETs’ performance. The GFET chips were washed with acetone for photoresist removal, soaked in ethyl acetate for 2 h for a better cleaning step, and, for electronic reading in a portable device, wire-bonded onto a printed circuit board (PCB). Transfer curves (TCs) were collected using PB of different concentrations (0.01; 0.1; 1; 10; 100 mM). On the other hand, to study the effect of pH on the performance of solution-gated graphene FETs, TCs were collected on a 10 mM PB buffer, and pH ranging from pH 5.0 to pH 9.0, where the pH is adjusted for each solution by adding small amounts of sodium hydroxide (NaOH) or hydrochloric acid (HCl).

#### 2.3.3. DNA Hybridization Detection with PB and PBS

**Figure 2:**
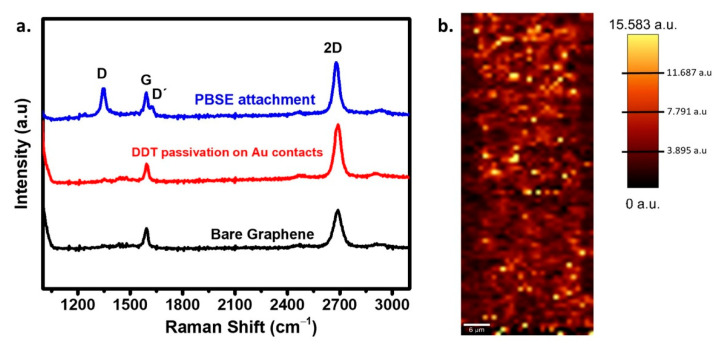
(**a**) Raman spectra of bare and modified graphene channel, (**b**) the corresponding 2D/G Raman intensity ratio maps of the single-layer graphene (SLG).

To optimize the DNA immobilization area on the graphene channel, the Au receded gate is passivated with 2 mM DDT in ethanol for 16 h (Figure 2a). Next, to facilitate the DNA immobilization onto the graphene, a 10 µL of 10 mM of PBSE in DMF is dropped onto the graphene channel area for 2 h, then consecutively washed with DMF and deionized water, followed by drying with N_2_ flow. The PBSE contains two binding sites; pyrene groups that attach to graphene through π−π interaction, and an ester group on the other side to capture the amine-tagged pDNA through NHS reaction. After modifying PBSE, a 10 µL of 10 µM pDNA is immobilized onto the PBSE modified graphene and kept in a humid chamber for 16 h at room temperature. To minimize the non-specific binding, a 10 µL of 10 mM ETA is added and stood for 30 min. A 10 µL of complementary tDNA is incubated for 1 h for the last stage’s hybridization process. The surface modification stages are illustrated in Figure 1b. The measurement of resistance is conducted in two buffers, PB (0.1, 1 and 10 mM) and PBS (0.01×, 0.1× and 1×) using the portable platform to obtain the TCs recording the drain-source current (I_DS_) under constant drain-source voltage (V_DS_) as a function of gate-source voltage (V_GS_).

#### 2.3.4. The Study of Probe DNA Adsorption Kinetic with PB and PBS in the Microfluidic System

The GFET sensor chip and a PDMS flow cell fitting the sensor layout are assembled and inserted in the portable platform (Figure 1a). This system is connected to a syringe pump and multi-position valves, allowing the automation and improvement of the volume and flow rate precision. The GFET chips have initially undergone the surface modification with DDT and PBSE linker. Accordingly, with the automatic micropump, a 1 mL of 10 µM pDNA is injected and flown into the sensor for a consecutive 16 h applying few stages of back and forward injections of 2 µL/s flow rate. The next step involves the blocking of the non-reactive linker site with ETA for 30 min. In the last stage, 1 mL of 1 µM of complementary tDNA is injected and flown into the sensor for one hour to hybridize with the pDNA. The ETA and tDNA solutions are applied in the same setting as was used for the pDNA immobilization. The buffer solution (PB or PBS) is then dispensed for the washing stage and signal measurement in between each step.

## 3. Results and Discussion

### 3.1. GFET Characterization and Surface Modification

As displayed on the sensor’s layout in Figure 1a, the liquid-sensing area of the GFET chip consists of a sensor array of nine graphene channels with a shared gate and an individually addressable source and drain. The optical microscope image shows one graphene channel with an area of 75 × 25 µm^2^ precisely flanked by two identical square Au source and drain large contacts covered with graphene, defining the channel length and providing an ohmic contact to the channel. In this way, efficient charge injection and collection from the channel are achieved in the biomolecular screening area even at low source-drain voltage.

PMMA is used as a temporary substrate for graphene transfer, which may generate unfavorable residual strain in graphene and leave contamination residues spread on the surface. After the cleaning procedure, Raman spectroscopy measurements show no traces of PMMA contamination demonstrated by the absence of the PMMA peak, typically observed at around 2900 cm^−1^ [27] in all the recorded spectra of the graphene channel (Figure 2a). Moreover, by averaging multiple Raman spectra at different positions on the sample’s surface, the graphene resulting from the PMMA assisted transfer onto the Si/SiO_2_ substrate exhibits the features of pristine monolayer graphene with negligible D peak at ~1351 cm^−1^, as well as a 2D (~2696 cm^−1^)-to-G (~1588 cm^−1^) intensity ratio (I_2D_/I_G_) of ~1.78. Subsequently, the Au surface blocking with DDT to eliminate the binding sites for the DNA/Au interaction was shown to have no impact on the monolayer graphene structure indicated by the spectral and intensity coincidence of the fingerprint peaks with those of the intact graphene. After PBSE functionalization, the I_2D_/I_G_ dropped to ~1.11, which is indicative of the disorder on the graphene surface concomitantly producing defects, as seen by the appearance of the D peak (~1351 cm^−1^). The D’ peak at ~1624 cm^−1^ emerging as a G peak shoulder is related to the graphene’s surface’s impurities and charges. This behavior is similar to that reported by Tsang et al., 2019, who chemically modified a graphene sensor for exosome identification [10]. The average shows the PBSE modified surface’s uniformity over three repetitions measured on different graphene channel sites (Figure S1, Supplementary Information (SI)). The high-quality graphene was validated by the 2D Raman mapping of the I_2D_/I_G_ ratio of dominant value of ~1.8–2.5 with a scanning area of ~2.3 mm^2^ (Figure 2b), showing a uniform coverage of the SLG over the chip.

### 3.2. The Effect of Ionic Strength and pH on the Graphene Transistor Response

**Figure 3:**
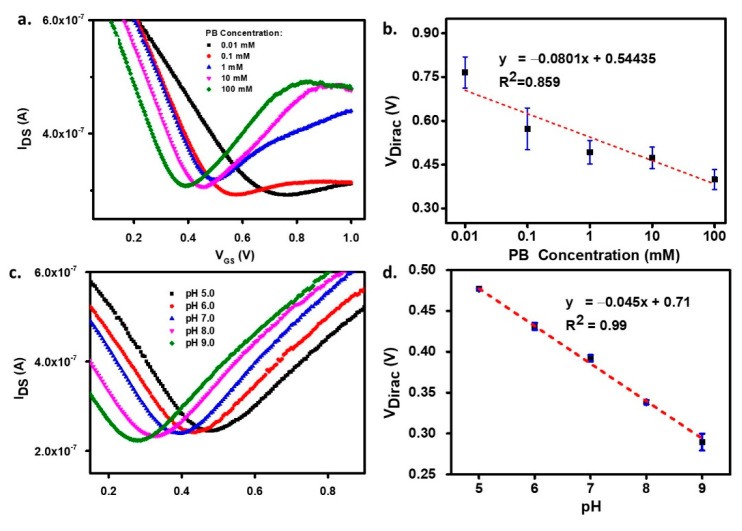
(**a**) I_DS_V_GS_ characteristic, and (**b**) extracted Dirac voltage point as the function of different phosphate buffer (PB) concentrations, (**c**) I_DS_V_GS_ characteristic, and (**d**) extracted Dirac voltage point as the function of varying pH.

The modulation of the channel conductance by the applied gate voltage is mediated by the capacitance of the interface graphene/liquid electrolyte, which depends on the solution’s ionic strength. But the ions and polar molecules that accumulate at the interface also have the effect of neutralizing any charges trapped at the graphene/silicon oxide substrate. Figure 3a,b display the GFET sensor’s response as a PB concentration function from 0.01 to 100 mM with a fixed pH of 7.00. The charts carry a wealth of information about the graphene sensor behavior. The first observation is that the graphene channel is unintentionally p-doped since all curves have the hole branch shifted to positive gate voltages. Moreover, Figure 3a shows that, while the hole branch of the TC is relatively insensitive to [PB] changes, the transconductance, *g_m_*, measured in the electron branch of the TC dramatically changes with [PB], from 0.49 to 0.43, 0.31, 0.10, and 0.08 µS at [PB] = 100, 10, 1, 0.1, and 0.01 mM, respectively. Assuming that changes in carrier mobility determine the observed *g_m_* changes, the I_DS_-V_GS_ curves imply that, while hole mobility is independent of ionic strength, the electron mobility of the graphene channel increases with higher ionic strength, with a considerably improved electron current regime at 1, 10, and 100 mM PB. This observation is consistent with the passivation by the buffer solution of charged defects and carriers (charge poodles) trapped at the graphene/substrate interface that act as scattering centers for the conduction electrons. At concentrations below 1 mM, PB could not provide adequate gate capacitance or trapped charge passivation for a high-mobility channel to form.

Another effect observable in Figure 3a,b is that increasing the PB’s ionic strength shifts the I_DS_-V_GS_ curves towards lower gate voltages. This observation agrees well with previously reported work [28] and is explained by the increased capacitance of the electrical double layers (*C_DL_*) formed at the graphene-electrolyte and electrolyte-gate interfaces. Since the Debye length (*λ_D_*) which sets the thickness of the EDLs, is proportional to the square-root of the inverse ionic strength of the solution (see Equation (1) and reference [5] herein), more concentrated PB solutions have smaller *λ_D_* and larger EDL capacitance, requiring less *V_GS_* to accumulate a given amount of charge in the channel. The Dirac point shift presented in Figure 3b is linearly dependent on log [PB] with a linearity of ~80 mV/decade [PB].

In interfacial sensing with GFETs, the conductance is mainly influenced by two concurrent mechanisms: Local gating by charged molecules in the vicinity of graphene that perturb the EDL and locally change the gate capacitance, and electron transfer, which may oppose the former effect, giving rise to opposite Dirac point shift directions [29]. In our experiment with pH, the non-modified graphene surface was in direct contact with the solution, with pH ranging from 3 to 9, which resulted in a shift toward lower voltage with increasing pH (Figure 3c). Our results suggest a charge transfer mechanism from the OH^−^ groups, generating n-type doping of graphene, raising the electrons’ chemical potential in graphene. Therefore, with the more electron-donating species (high pH), the TCs shift to lower *V_GS_*. We also believe that defective sites in graphene could assist in the chemisorption of the OH^−^ ions present in high concentration when the pH is above neutral, leading to charge tunneling onto the graphene and adding up to the charge transfer efficiency. The linear relation between Dirac point position and pH is depicted in Figure 3d with a ~45 mV/pH sensitivity.

### 3.3. The Effects of Salts in the Buffer and Buffer Dilution on DNA Hybridization Study

**Figure 4:**
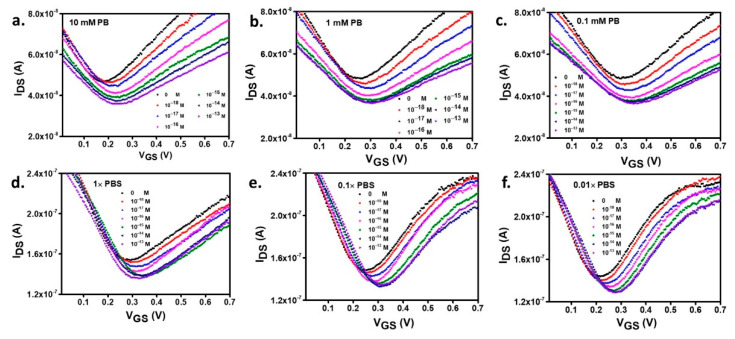
I_DS_V_GS_ curves of DNA hybridization detection using background solution of PB of (**a**) 10 mM, (**b**) 1 mM and (**c**) 0.1 mM and PBS (**d**) 1×, (**e**) 0.1×, and (**f**) 0.01×.

The selection of the liquid gate electrolyte plays an essential role in the overall performance of the GFET device, as discussed in the previous section. In a GFET biosensor, the electrolyte is typically a solution where the bio-compounds are maintained and is composed of buffer and salt species to keep the pH stable upon the presence of charged molecules in the solution. The counter ions in buffers influence the sensing mechanism in two ways: The adjustment of *λ_D_* for molecular binding events to occur inside the sensing interfacial volume [22] and the bulk electrolyte solution’s effect on the entire area of the interface. In this study, DNA hybridization detection is conducted in two types of commonly used buffers in biodetection work, PB and PBS, at a fixed pH of 7.4. Figure 4a–c represents the transfer curves after DNA detection in the PB background solution, with a 10, 1, and 0.1 mM concentration. A significant rise in the transconductance is observed in the transfer curve’s electron branch at the highest ionic strength (10 mM of PB), which agrees with the finding in Figure 3a where an enhanced electron mobility was observed for the higher ionic strength buffer. However, in the detection of target DNA from 1 aM to 100 fM, the Dirac point movement is more pronounced in the low ionic strength buffer ([PB] = 0.1 mM) plausibly due to the more favorable, longer Debye length (*λ_D_* = ~21 nm). In effect, comparing *λ_D_* with the size of the DNA molecule attached to the binder (*l_DNA + PBSE_* = ~10 nm), one sees that for high ionic strengths, the DNA duplex, which is more rigid than the single-stranded, becomes longer than the EDL (*λ_D_* = 6.65 and 2.11 nm for [PB] = 1 and 10 mM, respectively), thus decreasing the sensitivity because some hybridization part occurs outside the EDL area.

In DNA detection with the GFET, the Dirac point shifts to progressively higher voltages upon increasing the concentration of tDNA to hybridize the pDNA. Similar trends are observed in the detection using PBS buffer with 0.01×, 0.1×, and 1× dilutions, as seen in Figure 4d–f. The shifts result from the presence of the negatively charged oligonucleotides on the functionalized graphene surface. In DNA detection, the electrostatic gating mechanism is based on the modulation of *C_DL_* by the negatively charged DNA molecules that induce an equal amount of positive charge in the graphene channel, further lowering the electron chemical potential (Fermi level). Therefore a larger V_GS_ is required to raise the Fermi level into the conduction band, which occurs precisely at the Dirac point, as observed experimentally and is shown in Figure 4d–f.

The transfer curves acquired after each surface functionalization step reveal a common Dirac point shift direction, measured in both PB and PBS, after DDT (Au blocking), PBSE (linker attachment), ssDNA (probe immobilization), and ethanolamine (blocking), respectively (Figure S2). Using a PBS buffer electrolyte (Figure 4d–f), a higher carrier mobility is inferred from the higher values of *g_m_* for both the TC’s electron and hole branches. This fact emphasizes the salts’ effectivity in contributing to shielding charged impurities trapped in the SiO_2_ substrate and on the graphene surface [30,31]. On the other hand, additional amounts of small electrolyte counterions can insert themselves inter or intra-helically in DNA strands, provide ionic shielding, and reduce the repulsive force between DNA molecules, which enable the formation of tighter (zipped state) DNA double helices [32,33]. In DNA hybridization detection by Dirac point shift, a more prominent movement to higher voltage is observed in 0.01× PBS than at higher PBS concentrations, indicating the increase of sensitivity due to a larger volume of solution under space-charge effect, consequence of the longer Debye length, as discussed above. Additionally, the lower ionic strength with its reduced buffering capacity is essential in maintaining the solution’s pH value [34]. The complete list of the calculated Debye lengths is presented in Table 1.

**Figure 5:**
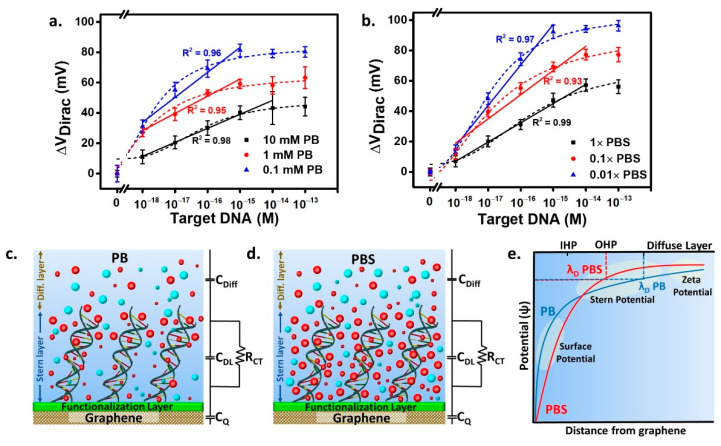
Calibration curves of DNA hybridization detection using a background solution of (**a**) PB, and (**b**) phosphate buffer saline (PBS) with R^2^ value corresponds to the linear fitting. Illustration of the ionic gradient on the interface with (**c**) PB, and (**d**) PBS backgrounds and (**e**) The schematic comparison of the electric potential at the interface from the measurement with PB and PBS. The large and small red symbols are positive ions with bigger sizes and smaller sizes, respectively. The large and small cyan symbols are negative ions with bigger and smaller sizes, respectively.

The calibration plots displayed in Figure 5a,b provide further insight into the TCs behavior shown in Figure 4. The dilution of the buffer and the consequent decrease of the ionic strength improved the GFET electrical performance in PB and PBS. A complete set of analytical parameters observed in this study, including the λ_D_, LoD, linearity, and sensitivity, are presented in Table 1. Under the DNA hybridization detection with PB, the highest PB concentration of 10 mM (λ_D_ of ~2.1 nm) provides the lowest sensitivity (9.22 mV/decade). In comparison, the highest sensitivity (16.47 mV/decade) is obtained in 0.1 mM PB (λ_D_ of ~21 nm). An extended dynamic range, one extra order of magnitude of concentration in the linear detection range of tDNA (10^−18^–10^−15^ M) than found in the detection with 10 mM PB (10^−18^–10^−14^ M), is also observed. Similar trends are found in the DNA hybridization detection with PBS. The lowest ionic strength (0.01× PBS) resulted in the highest sensitivity (26.69 mV/decade), which can be explained by the increase of Debye length. Nevertheless, in a comprehensive comparison of PB and PBS buffers, the Debye length is not the sole factor impacting the sensitivity. The measurement in 0.01× PBS (λ_D_ of ~7.6 nm) achieves ~62% higher sensitivity than that in 0.1 mM PB with higher Debye length (λ_D_ = ~21 nm).

The interfacial ionic density caused by additional salts’ presence in DNA detection is schematically depicted in Figure 5c,d. The total capacitance is estimated to be equal to 1/C_q_ + 1/C_DL_ + 1/C_Diff_ where C_q_ is graphene quantum capacitance [35], C_DL_ is the electrical double-layer capacitance, and C_Diff_ is the capacitance at the diffusion layer [36]. It is shown that with lower salts content, as in PB buffer (Figure 5c), the accumulation of charge tends to be on the upper part of the DNA molecule, and deep penetration by ions is likely limited. In contrast, in Figure 5d, the additional salts in PBS significantly contribute to the ionic density and ionic concentration in the Stern layer as well as, owing to the ability to penetrate deeper, until the inner Helmholtz plane (IHP) close to the sensor’s surface, especially for tiny sized ions. For the study of the charge crowding and penetration in both the PB and PBS models, one must also take into account the charge transfer resistance (R_CT_) in the Stern layer typically connected in parallel to the C_DL_ [37,38]. However, with the PBS background, the RCT is relatively small due to the predominance of C_DL_ resulting from the accumulation of small-sized counterions penetrating in between the DNA strands, while the opposite occurs in DNA detection with the PB background. The trade-off effects of the Debye length over sensitivity are modeled in Figure 5e. With the lesser ionic species in the PB background than in PBS, ions likely move in a considerably more spacious area, dominantly, outside the sites occupied by the DNA at the interface. Hence, the surface potential rises slower, eventually generating a longer Debye length. Measurement with additional salts in PBS, with higher counterions accumulation at the interface and within the DNA strands, dramatically leads to a higher potential close to the graphene channel than in PB. Close to the surface, the high potential results in the increase of C_DL_ with a faster potential rise resulting in a shorter Debye length than that occurring in measurement with PB.

The quantification of the limit of detection (LoD) of the DNA hybridization detection onto the GFET sensor under PB and PBS background is explained using the empirical model from the modified Hill’s function for the dose-response data. In the electrolyte gated FET configuration, the Debye length (λ_D_) is fundamentally linked to ionic strength, pH, and variation in the double-layer capacitance (C_DL_). Phosphate buffer (PB), made up of various ratio (R) of monobasic ($C_{2}^{0}$) to the dibasic ($C_{3}^{0}$) sodium phosphate salt has been widely applied in biological detection. However, the “physiological” isotonic version with around 300 mOsm/kg H_2_O, better known as phosphate buffer saline (PBS) has been widely used in various biomolecular detections such as proteins, lipids, oligonucleotides, and many more since its additional salts of NaCl and KCl may provide much improved in-vivo mimicking condition of an in-vitro ambiance. The concentration of the phosphate ions in PB can be predicted through the dissociation equilibrium below:

$$H_{3}{\mathrm{PO}}_{4}\overset{Ka1}{↔}H^{+}\;+\;H_{2}{{\mathrm{PO}}_{4}}^{-}\;H_{2}{{\mathrm{PO}}_{4}}^{-}\overset{Ka2}{↔}H^{+}\;+\;{\mathrm{HPO}}_{4}{}^{2-}\;{\mathrm{HPO}}_{4}{}^{2-}\overset{Ka3}{↔}H^{+}\;+\;{\mathrm{PO}}_{4}{}^{3-}.$$

The values of the equilibrium constants are pK*a1* = 2.12, pK*a2* = 7.21 and pK*a3* = 12.67 at 298 K respectively [39].

The ionic strength (*µ*) is calculated as follows:

$$\mu =\frac{1}{2}\sum CiZi^{2}.$$

In PB, it is calculated as:

$$µ=\frac{1}{2}\;(0C[H_{3}{\mathrm{PO}}_{4}]+\;1^{2}C[H_{2}{{{\mathrm{PO}}_{4}}^{-}}_{\;}]+\;2^{2}C[{\mathrm{HPO}}_{4}{}^{2-}{]}^{\;}+\;3^{2}C[{\mathrm{PO}}_{4}{}^{3-}{]}^{\;}+\;C[{\mathrm{Na}}^{+}])$$

while in PBS, the ionic strength includes the contribution from the NaCl and KCl shown below:

$$µ=\frac{1}{2}\;(0C[H_{3}{\mathrm{PO}}_{4}]\;+\;1^{2}C[H_{2}{{\mathrm{PO}}_{4}}^{-}{]}_{\;}+\;2^{2}C[{\mathrm{HPO}}_{4}{}^{2-}{]}^{\;}+\;3^{2}C[{\mathrm{PO}}_{4}{}^{3-}{]}^{\;}+\;C[{\mathrm{Na}}^{+}{]}^{\;}+C[K^{+}]).$$

The Debye length of both PB and PBS used in this study is calculated using the Debye-Hückel approximation [40]:

(1) $$\lambda_{D}=\;\sqrt{\frac{\varepsilon_{r}\varepsilon_{o}k_{B}T}{2N_{A}e^{2}µ}}$$

where $\varepsilon_{r}$ is the relative permittivity of water, $\varepsilon_{0}$ is the relative permittivity of vacuum, $k_{B}$ is the Boltzmann’s constant, *T* is temperature, $N_{A}$ is Avogadro’s constant, and *e* is the elemental charge.

For the statistical analysis for the DNA hybridization study, all the standard deviations in the calibration plots were taken from triplicate measurement on three individual sensors belonging to the same chip under identical experimental condition. The modified Hill’s function from Origin 9 software (OriginLab, Northampton, MA, USA) was applied to perform the non-linear regression of the recorded dose-response data with the fitting equation below:

(2) $$y=\;\frac{V_{min}+\left(V_{max}-V_{min}\right)}{1+\;\frac{10^{logEC50}}{10^{x}}}$$

where $V_{min}$ is the minimum DNA hybridization rate, $V_{max}$ is the maximum DNA hybridization rate, and EC50 refers to the concentration of pDNA that produces a half-maximal target DNA hybridization velocity.

To estimate the limit of detection (LoD) of the GFET sensor, we calculated two initial parameters [41] to be next used in the Equation (2) as follows:

(3) $$Limit\;of\;Blank\;\left(LoB\right)\;\;\;\;\;\;\;\;=\;mean_{blank}\pm 1.645\;\left(SD_{blank}\right)\;\;$$

(4) $$\;\;\;LoD_{\Delta V}\;\;\;\;\;\;\;\;\;\;\;\;\;\;\;\;\;\;\;\;\;\;\;\;\;\;\;\;\;\;\;\;\;\;\;=LoB\;\pm 1.645\;\left(SD_{lowest\;concentration}\right).$$

Table 1 lists all the analytical parameters in the DNA hybridization detection using the fabricated GFET sensor with different background buffers.

The modified Hill’s function has been reported to fit well the correlation of the biosensor’s response to the binding activity [41]. We found out that the increased sensitivity obtained by lowering the electrolyte’s ionic strength was positively correlated to lower LoD attainment in the DNA hybridization detection, a consequence of the low standard deviation in the blank measurement. It can be explained by the low content of counterions confined at the nanoscale interface system with lower ionic strength. Hence, during the blank measurement, the charge perturbation was much less than that measured in the high ionic-strength electrolyte. Comparing the effect of the salts in PB and PBS background, despite achieving the same order magnitude of LoD value, the salts in the measurement with PBS resulted in larger LoD value than under PB screening, which indicates the high interference of salts in Stern layer during the blank testing causing higher standard deviation. The findings show that despite the sensitivity being not directly affected by the Debye length, the Debye length is closely related to LoD, which relies on the blank sample measurement signal.

### 3.4. The Effects of Salts in the Buffer on DNA Adsorption Kinetics

**Figure 6:**
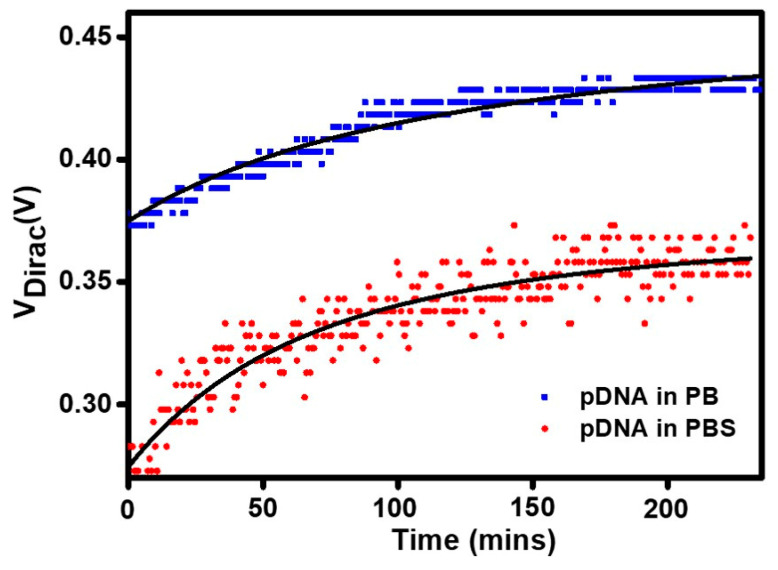
Adsorption kinetics curves (solid squares and circles) of the probe DNA immobilization on functionalized Graphene Field-Effect Transistors (GFET) sensor surface measured in two different electrolyte backgrounds. The solid lines are fittings to the Langmuir isotherm.

In various DNA adsorption setups onto a sensor, the association constant significantly depends on the salts’ concentration [42]. To comprehend additional salts’ impact in real-time DNA adsorption kinetics, we introduced the single-stranded probe DNA onto the PBSE modified graphene channel using the microfluidic system under different electrolytes. In the adsorption curves in Figure 6, Dirac voltage points represent the amount of the adsorbed pDNA, which increases with incubation time. The curves were obtained from the Dirac voltage position recorded from the start of probe DNA injection to the system until the saturation point was reached at intervals of 240 min. The curves show a non-linear behavior as fitted by the Extended Langmuir adsorption isotherm equation using Origin 9 software (OriginLab, Northampton, MA, USA) with the parameters shown in Table 2. The following equation (Equation (5)) fits the experimental data for both pDNA adsorption measured in PB and PBS background.

(5) $$y=\;\frac{a.bx^{\left(1-c\right)}}{1+bx^{\left(1-c\right)}}.$$

In this equation, *y* refers to the dependent parameter, the Dirac point voltage, which is equivalent to fractional coverage (*θ*), typically used in the Langmuir adsorption isotherm realm. The *x* refers to the incubation time, and *b* is equal to the equilibrium constant *K* (*k_d_/k_a_*) [43,44], where *k_a_* and *k_d_* are the rate constants for adsorption and desorption, respectively. In this experiment, we focused on the immobilization of the probe DNA molecule; hence, the *k_d_* is considered the same value in both background electrolytes. With a constant number for *k_d_*, it is estimated that based on the obtained fitting variables, the pDNA adsorption, *k_a_*, in PBS background is greater than that in the PB (shown by the smaller value of variable b for PBS), indicating faster isotherm properties.

Figure 6 shows that in a dynamic setup, in PBS, the saturation point of the pDNA immobilization is achieved at around 200 min, while in PB, stability appears slightly later. The contrast is seen in the initial stage of pDNA absorption, demonstrating a rapid increase in V_Dirac_ under PBS screening. This finding aligns well with the ionic gradient’s impact, penetration, and the electric potential explained in Figure 5c–e. A large amount of additional multivalent ions in PBS also results in the more scattered data points taken from a similar surface area than that in the measurement with PB background with less and sparser ionic activity at the interface. Furthermore, the additional salts in PBS provide more intense electrostatic shielding around the pDNA, facilitating better adsorption. In the previously reported works, the hydrated state of sodium ions enabled the direct interaction with an oxygen atom in the nucleotide or via hydrogen bonding with metal-coordinated water molecules [45]. Not only that, but the intensified adsorption in PBS could also be yielded by the more compact structure of the effective DNA diameter on the surface due to the occurrence of the neutralization events between the cations from PBS and the negative DNA charges [32,33,46].

## 4. Conclusions

A series of interfacial ionic interactions on liquid-gated GFET sensors, operated in a miniaturized and portable DNA screening system, is described in detail using different background electrolytes’ employment. The ionic strength adjustment was the most critical stage in applying the GFET sensor. The single-layer graphene structure in a FET channel is sensible to pH change, due to a charge transfer mechanism. A distinct behavior of PB and PBS, the prevalently used background buffers in bio-detection, along with the impact of the dilution, was recorded. Carried out in DNA hybridization detection, both PB and PBS demonstrated the dilution’s effectivity in improving sensitivity and dynamic range. However, a significant finding is that Debye length is not the sole factor in the GFET device performance. Despite the longer estimated Debye length achieved in PB background, the sensitivity is not higher than in PBS background. The additional salts in PBS increase graphene carrier mobility due to more efficient charge impurity passivation by counterions at the interface than in PB. These crowding ions may penetrate more in-depth to the Stern layer at the sensor’s surface, significantly affecting the total surface potentials and reinforcing the DNA charges’ electrostatic shielding. The additional salts in PBS also accelerated the isotherm kinetics of the oligonucleotides adsorption onto the surface. The choice for a precise optimum buffer concentration is indeed different for different molecular targets due to the biomolecules’ intrinsic chemical functions. Here, some guidelines for the selection of the most suitable buffers were established.

## Figures and Tables

**Table 1:** Analytical parameters of DNA detection on GFET sensor with different background buffers.

Buffer.	*µ* (mM)	*λ_D_* (nm)	Hill’s Function						Linear Area Fitting		
			*R* ^2^	*SD* *Blank*	*LoB*	*SD* *Lowest Conc.*	*LoD* Δ*V*	*LoD* (*aM*)	*R* ^2^	Linear Region (M)	Sensitivity (mV/dec.)
0.1 mM PB	0.1	21.00	0.98	0.0054	0.0088	0.0042	0.015	4.17	0.96	10^−18^–10^−15^	16.47
1 mM PB	1	6.65	0.96	0.0052	0.0090	0.0029	0.013	3.44	0.95	10^−18^−10^−15^	11.30
10 mM PB	10	2.11	0.89	0.0022	0.0037	0.0044	0.011	10.15	0.98	10^−18^–10^−14^	9.22
PBS 0.01×	1.627	7.61	0.99	0.0010	0.0016	0.0027	0.006	5.01	0.97	10^−18^–10^−15^	26.69
PBS 0.1×	16.27	2.41	0.97	0.0019	0.0031	0.0034	0.008	7.58	0.93	10^−18^–10^−14^	15.95
PBS 1×	162.7	0.76	0.95	0.0026	0.0042	0.0037	0.010	22.60	0.99	10^−18^–10^−14^	12.67

*µ* is the ionic strength of the buffer, *λ_D_* the Debye length, *R*^2^ is the correlation coefficient of the respective fitting, *SD Blank* is the standard deviation of the average blank points, *LoB* is the limit of blank, *LoD* Δ*V* is LoB ± 1.645 × (standard deviation of the lowest concentration signal), *LoD* is the limit of detection.

**Table 2:** The pDNA adsorption kinetic variables measured in different electrolytes.

Background Solution	Variables			Regression Coefficient
	a	b	c	
PB	0.468	0.049	0.044	0.972
PBS	0.372	4.124 × 10^−4^	−0.916	0.881

## Supplementary Materials

The following are available online at https://www.mdpi.com/2079-6374/11/1/24/s1, Figure S1: The reproducibility of the PBSE modified surface shown by the average over 3 repetitions measured on (a) different sites of the graphene channel recorded by an optical microscope and (b) the collected Raman spectra., Figure S2: I_DS_V_GS_ curves of each stage of surface functionalization on the GFET sensor measured using background solution of PB of (**a**) 10 mM, (**b**) 1 mM, and (**c**) 0.1 mM and PBS (**d**) 1×, (**e**) 0.1×, and (**f**) 0.01×.
