# Supplementary material for: Influence of the Electrolyte Salt Concentration on DNA Detection with Graphene Transistors
Source: Biosensors (Basel). 2021 Jan 17;11(1):24. doi: 10.3390/bios11010024 (PMC7830926; doi:10.3390/bios11010024)
Supplement: Supplementary file 1 [file biosensors-11-00024-s001.pdf]

Supplementary Information

Influence of the electrolyte salt concentration on DNA detection with graphene transistors

Agnes Purwidyantri[1,*], Telma Domingues[2], Jérôme Borme[2], Joana Rafaela Guerreiro[1], Andrey Ipatov[1], Catarina M. Abreu[3,4], Marco Martins[5], Pedro Alpuim[2,6,*], Marta Prado[1]

[1] Food Quality and Safety Group, International Iberian Nanotechnology Laboratory (INL), Av. Mestre José Veiga, 4715-330 Braga, Portugal; agnes.purwidyantri@inl.int (A.P.); joana.guerreiro@inl.int (J.R.G.); andrey.ipatov@inl.int (A.I.); marta.prado@inl.int (M.P.)

[2] 2D Materials and Devices Group, International Iberian Nanotechnology Laboratory (INL), Av. Mestre José Veiga, 4715-330 Braga, Portugal; telma.domingues@inl.int (T.D.); jerome.borme@inl.int (J.B.); pedro.alpuim.us@inl.int (P.A.)

[3] Nanomedicine Group, International Iberian Nanotechnology Laboratory (INL), Av. Mestre José Veiga, 4715-330 Braga, Portugal

[4] Current Address: 3B's Research Group, I3Bs – Research Institute on Biomaterials, Biodegradables and Biomimetics, University of Minho, AvePark, Parque de Ciência e Tecnologia, Zona Industrial da Gandra, 4805-017 Barco, Guimarães, Portugal; catarina.abreu@i3bs.uminho.pt (C.M.A.)

[5] Nano-ICs Group, International Iberian Nanotechnology Laboratory (INL), Av. Mestre José Veiga, 4715-330 Braga, Portugal; marco.martins@inl.int (M.M.)

[6] Center of Physics, University of Minho, 4710-057 Braga, Portugal

[*] Correspondence: agnes.purwidyantri@inl.int (A.P.); pedro.alpuim.us@inl.int (P.A.)

The surface functionalization with PBSE before the pDNA immobilization step showed new peaks as compared to the bare graphene and Au contacts blocking with DDT, namely D′and D peaks. The PBSE does not alter the intrinsic properties of the graphene since the pyrene group constituting PBSE π-π stacks with graphene to form the self-assembled monolayer with uniform coverage, whereas, the N-hydroxysuccinimide (NHS) binds the amine groups tagging the DNA [1]. The homogenous coverage of the PBSE linker is observed from different spots of measurement (Figure S1 a) in the following Raman spectra taken (Figure S1 b).

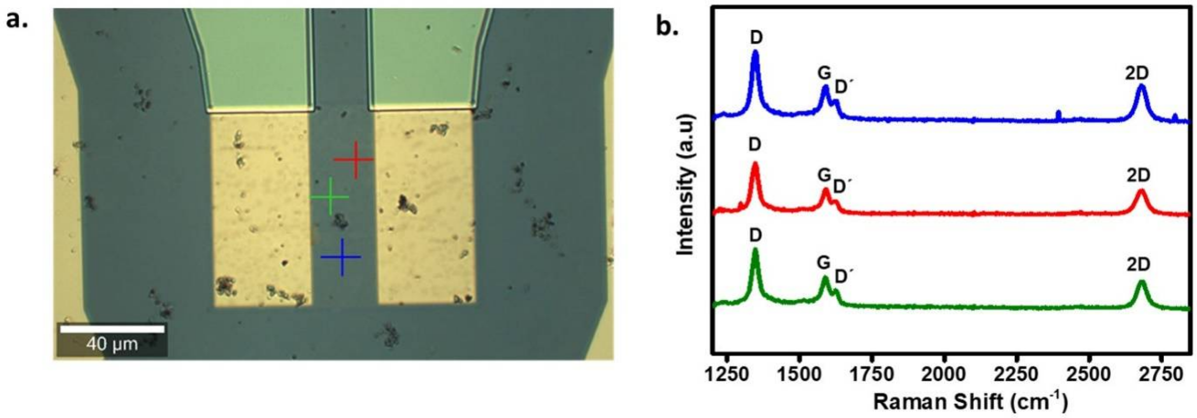

Figure S1. The reproducibility of the PBSE modified surface shown by the average over 3 repetitions measured on (**a**) different sites of the graphene channel recorded by an optical microscope and (**b**) the collected Raman spectra.

Figure S2 represents the surface functionalization effects on graphene transfer curves. The initial stage of surface functionalization was the blocking of Au contact surface with DDT, which governs the dipole formation on the interface. The DDT contributes to a sum of positive charge onto the interface and was likely cleaning the graphene surface from impurities and thus, generates a shift towards lower gate voltage. Subsequently, the addition of PBSE linker enhances the p-doping on the graphene due to the π-π interaction [2], [3]. Hence, a positive shift in the transfer curves was observed. In the pDNA immobilization, successful confinement of net negative charge from the phosphate backbone DNA results in the acquisition of higher voltage, which shifts the curves in the right direction. In the last functionalization stage, ETA was added to eliminate the non-reactive NHS-ester ligands as well as the weakly immobilized pDNA on the PBSE. Therefore, a shift towards lower gate voltage in the transfer curves was recorded to compensate for the reduction of net negative charge on the interface. The trends of the transfer curves shift are identical in both PB (Figure S2 a-c) and PBS groups (Figure S2 d-f). Contrasts are seen in the higher electron mobility in the PBS than in the PB group implying the role of the crowded counterions from salts in PBS speeding up the molecular interfacial interaction. We also observed that in the surface functionalization with PBS, the lower ionic strength demonstrates more facile electron mobility as depicted in the sharpness of the transfer curves.

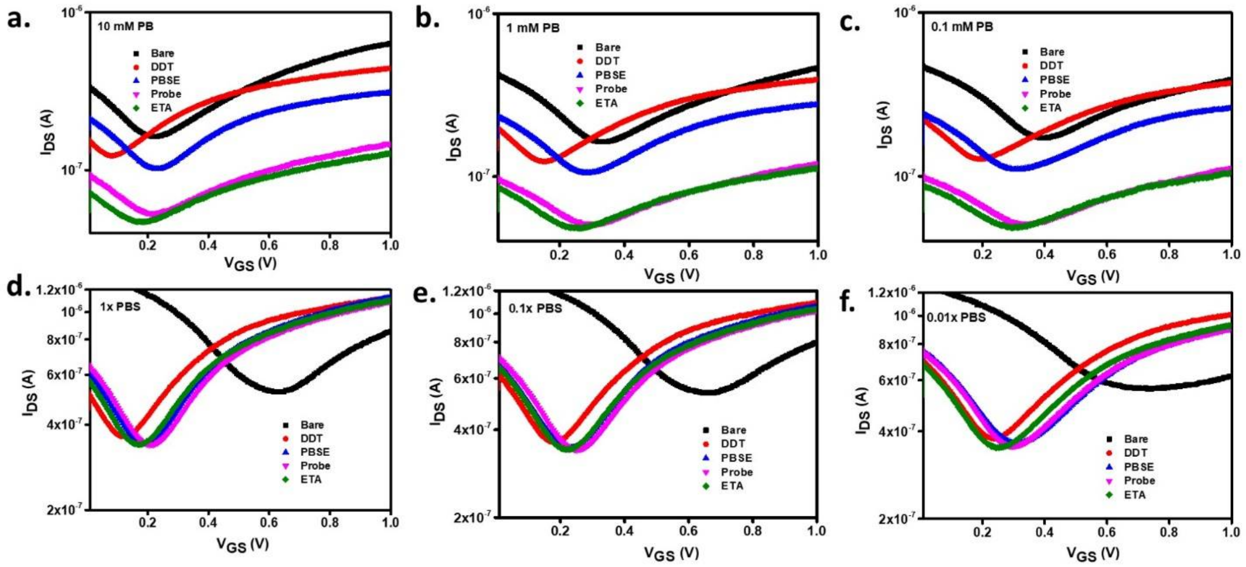

Figure S2. $I_{DS}V_{GS}$ curves of each stage of surface functionalization on the GFET sensors measured using background solution of PB of (**a**). 10 mM, (**b**). 1 mM and (**c**) 0.1 mM and PBS (**d**). 1×, (**e**). 0.1×, and (**f**). 0.01×.

References

[1] R. Campos *et al.*, "Attomolar label-free detection of dna hybridization with electrolyte-gated graphene field-effect transistors," *ACS Sensors*, vol. 4, no. 2, pp. 286–293, 2019.

[2] R. Forsyth, Anitha Devadoss, and Owen J. Guy, "Graphene Field Effect Transistors for Biomedical Applications: Current Status and Future Prospects," *Diagnostics*, vol. 7, no. 3, p. 45, 2017.

[3] J. Ping, R. Vishnubhotla, A. Vrudhula, and A. T. C. Johnson, "Scalable Production of High-Sensitivity, Label-Free DNA Biosensors Based on Back-Gated Graphene Field Effect Transistors," *ACS Nano*, vol. 10, no. 9, pp. 8700–8704, 2016.
